# Pulsed laser-induced formation of silica nanogrids

**DOI:** 10.1186/1556-276X-9-102

**Published:** 2014-03-02

**Authors:** Jürgen Ihlemann, Ruth Weichenhain-Schriever

**Affiliations:** 1 Laser-Laboratorium Göttingen, Hans-Adolf-Krebs-Weg 1, Göttingen 37077, Germany

**Keywords:** Laser, Ablation, Silica, Silicon suboxide, Nanogrid, Confinement

## Abstract

Silica grids with micron to sub-micron mesh sizes and wire diameters of 50 nm are fabricated on fused silica substrates. They are formed by single-pulse structured excimer laser irradiation of a UV-absorbing silicon suboxide (SiO_*x*_) coating through the transparent substrate. A polydimethylsiloxane (PDMS) superstrate (cover layer) coated on top of the SiO_*x*_ film prior to laser exposure serves as confinement for controlled laser-induced structure formation. At sufficiently high laser fluence, this process leads to grids consisting of a periodic loop network connected to the substrate at regular positions. By an additional high-temperature annealing, the residual SiO_*x*_ is oxidized, and a pure SiO_2_ grid is obtained.

**PACS:**

81.07.-b; 81.07.Gf; 81.65.Cf

## Background

There are a lot of approaches to treat substrate-bound thin films by pulsed lasers in order to modify the structure, morphology, or functionality of these layers. Either the internal physical or chemical properties are modified maintaining the external shape (annealing, crystallization, transformation), a well-known example of which is the crystallization of amorphous silicon on glass for display applications [1], or the external morphology is changed, which is the case, e.g., for dewetting [2] or (partial) ablation. Patterning of thin metallic, semiconducting, or dielectric films by laser ablation has been extensively studied, and numerous applications utilizing this method have been developed [3]. There are also ablation processes aimed at spatially selective deposition of material on another substrate, this process being named laser-induced forward transfer (LIFT) [4]. If the ablation/transfer is incomplete in that sense that the layer detaches from the substrate in some area, but the film is still not perforated, blister formation is observed [5].

In this paper, we describe a method utilizing the space-selective laser-induced film detachment together with some morphology change due to heating and surface tension to create substrate-bound grid structures with micron to nanometer dimensions. The fabrication of such grids from silica material relies on the combination of two fundamental conditions of laser ablation.

First, effective and controlled material response is possible only if the laser radiation is strongly absorbed by the treated material. As well-controlled absorption of laser light in silica (SiO_2_) is impeded by the transparency of this material, we choose highly absorbing silicon suboxide (SiO_*x*_, *x* ≈ 1) as primary material for laser treatment, which can be oxidized to SiO_2_ after the laser-induced shape-forming process [6].

Second, shape control in laser ablation is strongly enhanced by the so-called confinement. A liquid or a polymer layer in contact with the surface to be ablated serves for smooth, contiguous bulges around the ablation holes instead of irregular splashes observed without this confinement [7]. In standard ablation configurations, this confinement material has to be transparent for the laser radiation, because the laser beam has to pass it before being absorbed at the surface of the material to be ablated. Therefore, it is preferably applied in the form of thin layers. Using a rear side configuration, where the beam is guided through the substrate onto the film [8], this transparency is not that critical, i.e., thick layers can be used for confinement.

**Figure 1:**
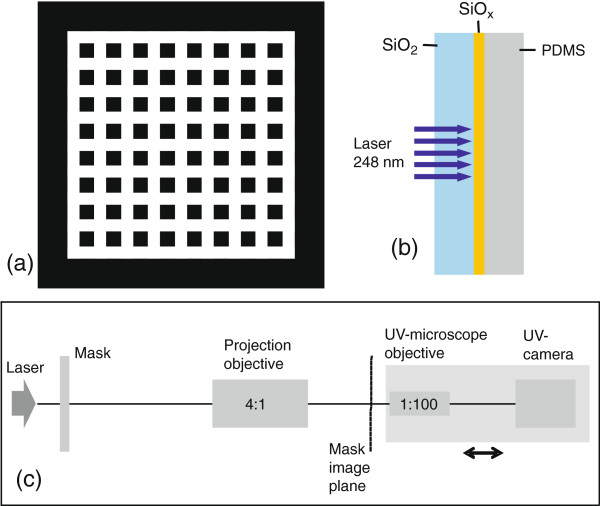
**Experimental arrangement.** Mask design with transparent stripes (white) **(a)**, sample configuration for laser processing **(b)**, and experimental arrangement for mask projection and for the measurement of the beam profile in the sample plane (=mask image plane) **(c)**.

Our approach is the following: a thin SiO_*x*_ film (thickness < 200 nm) is deposited on a fused silica substrate, and a thicker polydimethylsiloxane (PDMS) confinement film (several microns to millimeters thick) is casted or spin-coated on top of it (Figure 1b). A nanosecond KrF or ArF excimer laser (wavelength 248 or 193 nm, respectively) is used for single-pulse irradiation of the SiO_*x*_ film through the transparent substrate, selecting a spatially periodic intensity pattern (Figure 1a). The thin SiO_*x*_ film absorbs the laser radiation and, at sufficiently high fluence (laser pulse energy per irradiated area), forms blisters at the intensity spikes under the confinement of the covering soft polymer material. Increasing the laser fluence - depending on this fluence, the spatial intensity distribution, and the SiO_*x*_ film thickness - the film softens, stretches, tears, and resolidifies in a well-controlled way so that a regular meshwork or grid pattern is formed. After removing the PDMS, this grid, which is still connected to the substrate, can be oxidized to silica by a high-temperature annealing process in air.

## Methods

SiO_*x*_ films of 20- to 200-nm thickness with *x* ≈ 1 were deposited on 2-mm-thick fused silica substrates by vacuum evaporation (Laseroptik, Garbsen, Germany). These coatings are hard, exhibit good adhesion, and are chemically stable at room temperature. In contrast to SiO_2_, they absorb strongly in the ultraviolet spectral range. The absorption coefficient of SiO_*x*_ at 248 nm is about 2.7 × 10^5^ cm^−1^ for *x* ≈ 1, and the refractive index is about *n* = 1.9 [9]. A 2-mm-thick film of PDMS (Sylgard 184, Dow Corning, Midland, MI, USA) was casted over the SiO_*x*_ coating and dried in air at room temperature.

Irradiation experiments were carried out using a standard KrF excimer laser emitting at 248 nm with pulse duration of about 25 ns. The laser illuminates a mask, which is projected on the sample with × 4 demagnification using an objective with a numerical aperture of NA = 0.13 (4x/10-248, MicroLas, Göttingen, Germany). Illuminating mask fields of 5 mm × 5 mm size homogeneously, sample areas of 1.25 mm × 1.25 mm can be treated with a single exposure. Crossed grating Cr-on-quartz masks with various periods *p* were used (Figure 1a). They consist of transparent stripes of width *p*/2 with pitch *p* in two orthogonal directions, corresponding to an array of opaque Cr squares with side length *p*/2 and pitch *p*.

The fluence was determined by measuring the total energy arriving in the sample plane divided by the whole illuminated field. If this image field has the size *S* and the mask pattern is correctly imaged, the effectively illuminated area amounts to 0.75 × *S* because of the Cr fill factor of 0.25, so that the local fluence in the maxima is actually a bit higher. All irradiation experiments have been performed in a rear side configuration, i.e., the beam is directed through the fused silica substrate onto the SiO_*x*_ film (Figure 1b).

To determine the intensity distribution in the image plane on the sample, the sample is removed, and this plane is imaged onto a UV-sensitive CCD camera using a × 100 UV microscope objective (Ultrafluar, Carl Zeiss, Oberkochen, Germany) (Figure 1c).

Irradiation experiments with high spatial resolution were carried out using a standard ArF excimer laser emitting at 193 nm with pulse duration of about 20 ns. In this case, a Schwarzschild-type reflective objective (NA = 0.4, ×25 demagnification) was used for mask projection. A scanning electron microscope (Zeiss DSM 962) has been used to investigate the laser-induced morphological changes.

## Results

**Figure 2:**
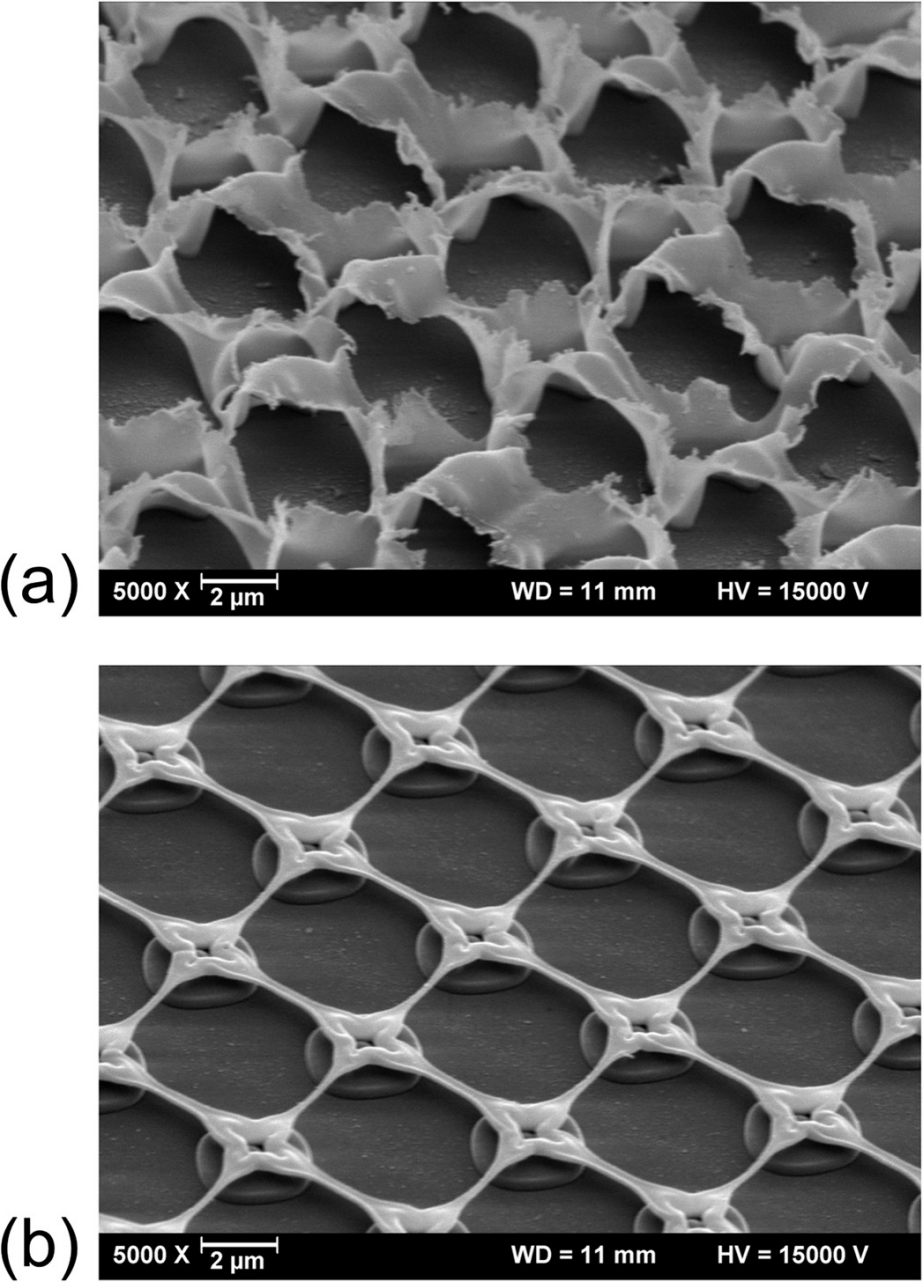
**Influence of confinement.** Patterned 150-nm-thick SiO_*x*_ film irradiated **(a)** without and **(b)** with confinement (after peeling off the confinement layer); laser parameters: 248 nm, 260 mJ/cm^2^, 1 pulse.

Figure 2 displays SiO_*x*_ films irradiated with a crossed grating pattern with and without PDMS confinement layer (after peeling off this layer). In both cases, the film disintegrates with a period given by the beam pattern, whereas the fused silica substrate remains intact. Confinement leads to smooth, contiguous features around the ablation sites instead of irregular splashes observed without this confinement.

**Figure 3:**
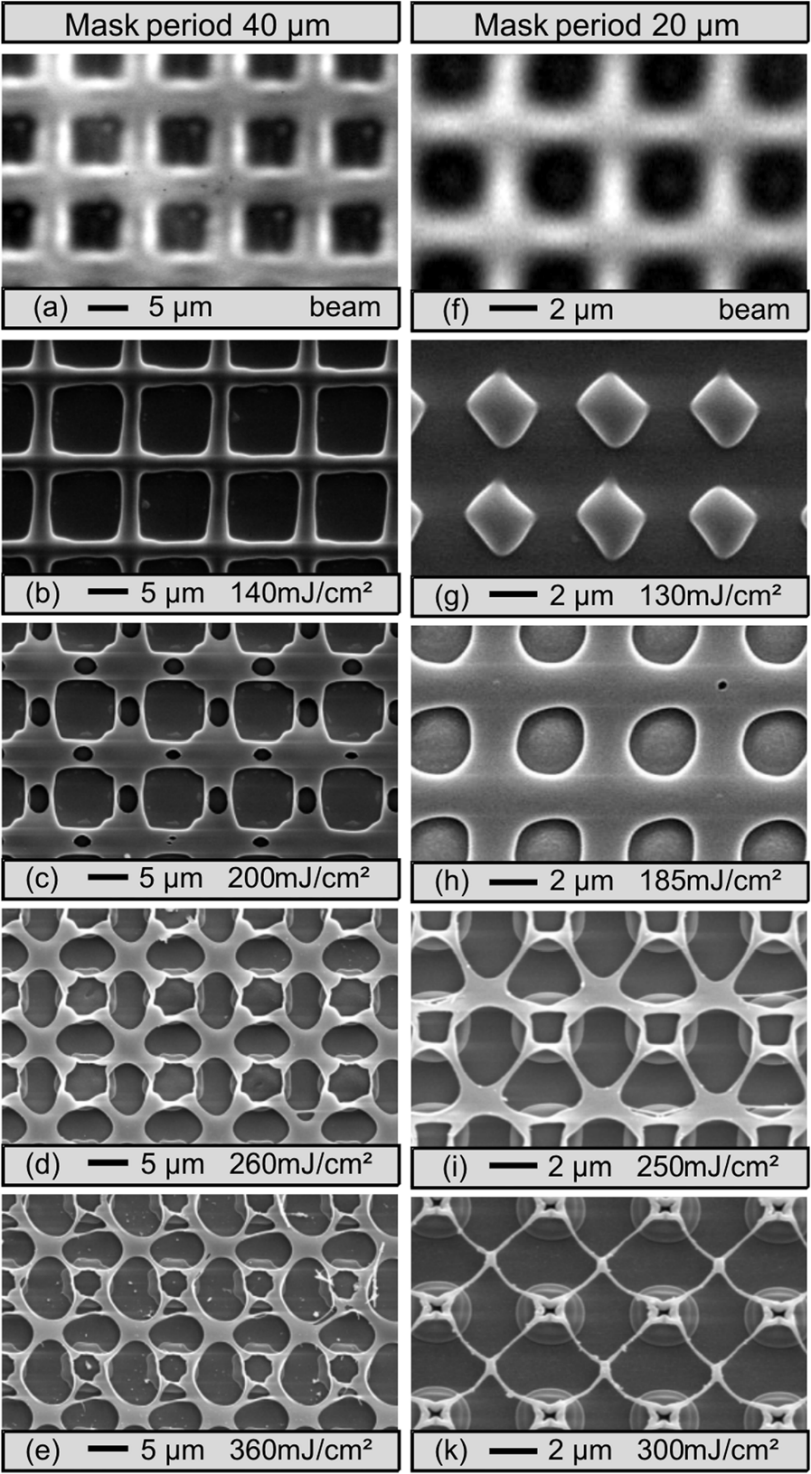
**Fluence dependence of the structure formation.** Intensity distribution in the sample plane **(a, f)** (contrast enhanced for clarity) and corresponding patterns in 150-nm-thick SiO_*x*_ films obtained with single pulses of varying fluences at 248 nm, mask period 40 μm **(b to e)**, and mask period 20 μm **(g to k)**.

To establish a correlation between the irradiation pattern and the resulting grid pattern, beam profiles in the sample plane have been recorded (Figure 3). In the case of a large period of the mask (40 μm), the intensity pattern is a four times reduced, but congruent, image of the transmission pattern of the mask (a). In the case of the 20-μm mask period, the beam pattern is already a bit blurred due to the limited resolution of the projection optics (f). The corresponding grid patterns obtained at various fluences are also displayed in Figure 3. At low fluence, in the case of a period large compared to the optical resolution, the film detaches from the substrate in the area of the irradiated cross pattern forming hollow channels, but keeping contact to the substrate in the non-irradiated areas (b). For the smaller period, only some buckling of the film at the high intensity crossing points is observed (g). Increasing the fluence, after enlargement of the detached area (c, h), rupture of the film in between the crossing points of the channels and formation of openings in the detached film occur (d, i). At still higher fluence, the enlargement of the openings (e) and the formation of thin wires of residual material between these openings (k) are observed. However, at the positions of minimum intensity, this wire grid is still connected to the substrate. Depending on the fluence and the particular intensity pattern, other types of shaping can be observed, e.g., hollow channels or arrays of blisters or cup-like structures.

**Figure 4:**
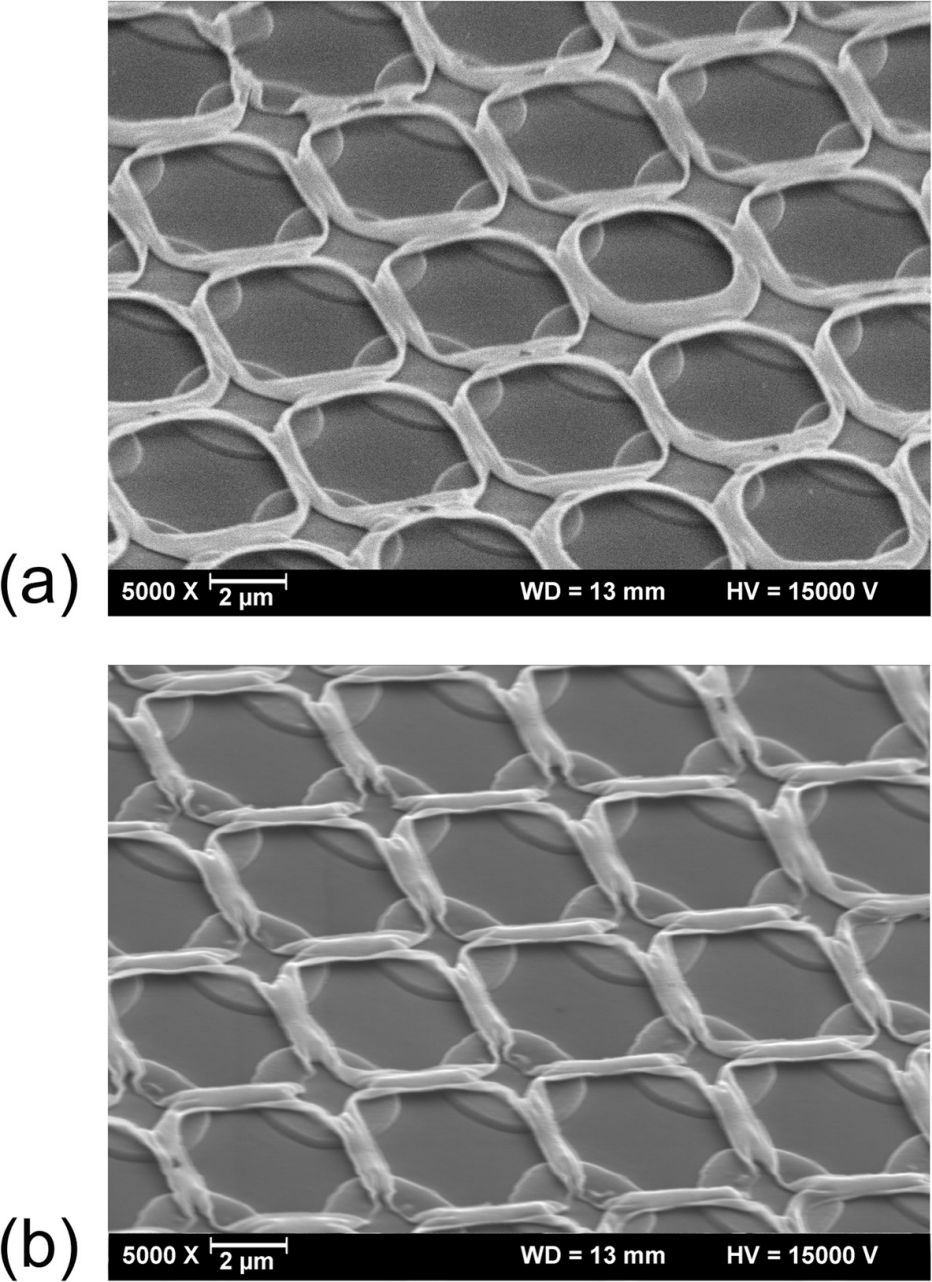
**Pattern before and after annealing.** Grid pattern generated in a 90-nm-thick SiO_*x*_ film at 248-nm laser wavelength: **(a)** 185 mJ/cm^2^, before annealing; **(b)** 210 mJ/cm^2^, after oxidation to SiO_2_ by high-temperature annealing (1,273 K, 16 h).

By heating the sample to >1,000 K, the material is oxidized to SiO_2_ leading to a chemically even more stable silica wire grid (Figure 4).

**Figure 5:**
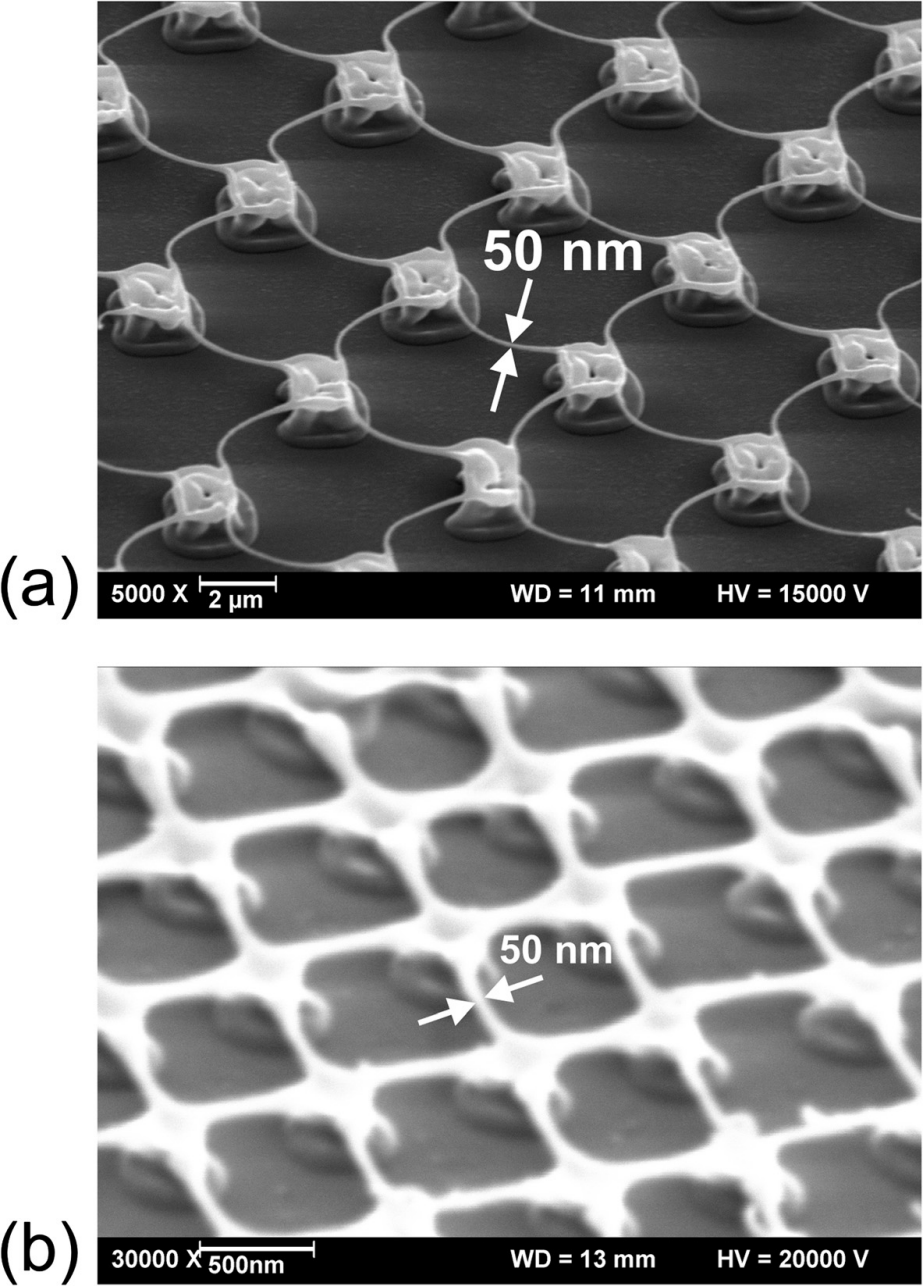
**Grids with wire diameters at the nanoscale. (a)** Grid pattern generated in a 144-nm-thick SiO_*x*_ film using a laser wavelength of 248 nm and a fluence of 300 mJ/cm^2^. **(b)** Grid pattern generated in a 28-nm-thick SiO_*x*_ film using a laser wavelength of 193 nm and a fluence of 130 mJ/cm^2^.

Grids with periods from the sub-micron range to more than 10 μm have been fabricated by this method. The particular final shape depends on the irradiation pattern, the fluence, and the film thickness. Figure 5 displays grids with wire diameters of about 50 nm. In Figure 5a, the nanowires bridge a distance of 5 μm, so that the length/diameter ratio amounts to 100:1. Figure 5b demonstrates that nanogrids with a sub-micron mesh width (800 nm) can be made. In this case, the self-supporting wires have a diameter of 50 nm, too.

## Discussion

The method utilizes the combination of pulsed laser heating and softening of a thin film, expansion, fracture and shaping due to pressure generation and surface tension, and resolidification in the final shape. It shows that a pulsed laser forming process is possible that delivers reproducible patterns, which depend on the irradiation pattern, but do not directly reproduce the mask or irradiation pattern.

The forming of films in the described way is possible for film thickness below about 200 nm. For thicker films, a transfer process of intact film pads is observed instead [10]. It is assumed that for the grid-forming process complete melting of the film is necessary, but vaporization must be limited to an extent, that the remaining molten material can be formed by the shock wave generated by this vaporization in combination with surface tension. Regarding the optical absorption depth and the thermal diffusion length for the given laser and material parameters, 200 nm corresponds to a maximum depth to which the melting temperature can be reached without excessive boiling [11].

Assuming that the final topographies for low or medium fluence represent intermediate states of the process at high fluence, the formation of a nanogrid array can be understood as follows: The blister formation starts at the points of maximum intensity. Some time later, the heated film is elevated in the whole irradiated area and is connected to the substrate only at the border of the remaining non-irradiated spots in between. After further expansion, this elevated (molten) film breaks open and reshapes under the influence of surface tension. The formation of the wire grid with closed loops is completed by the constriction of this perforated film into thin wires with anchor points on the unaffected film pads on the substrate. Depending on the specific irradiation pattern and the resulting positions of film rupture, nodes of the wires in between these anchor points above the substrate level are formed.

In contrast to the so-called laser dynamic forming (LDF) [12], the shape of the resulting structure is not determined by the shape of a mold, but only by the beam pattern and the material parameters of film and confinement layer. However, in some cases, LDF utilizes a polymer encapsulation of the film to be formed to minimize degradation of the functional film in a similar way to the polymer confinement of this work [13].

## Conclusion

Silica wire grids with micron- to sub-micron-size periods and nanometer wire diameter are made by patterned laser irradiation of silicon suboxide films on quartz substrates with polymer top confinement. The specific grid pattern can be varied by tuning fluence and irradiation pattern. The process is based on pulsed laser-induced local softening, forming, and resolidification under control of the confinement layer. Various applications in the fields of optics, micro- and nanofluidics, or medical technology (adhesion of cells) are imaginable.

## Competing interests

The authors declare that they have no competing interests.

## Authors’ contributions

JI conceived of this study and drafted the manuscript. RW-S performed the laser experiments and the SEM analysis. Both authors evaluated the results and read and approved the final manuscript.
